# Histopathological spectrum of primordial odontogenic tumor with co-existing dentigerous cyst: 1^st^ reported case of the world with a proposed ‘updated diagnostic criteria’

**DOI:** 10.1186/s13000-024-01560-8

**Published:** 2024-10-29

**Authors:** Dhara Dwivedi, Nitin Prabhakar, Monal Yuwanati, Gunjan S. Aswal, Renu Rawat

**Affiliations:** 1 Department of Dental Medicine, College of Medicine and Health Sciences, Hawassa University Comprehensive Specialized Hospital, Hawassa, Ethiopia; 2 Department of Pathology, Saveetha Dental College and Hospitals, Saveetha Institute of Medical and Technical Sciences, Saveetha University, Chennai, 600077 India; 3 Department of Dental Medicine- Oral & Maxillofacial Surgery Division, College of Medicine and Health Sciences, Hawassa University Comprehensive Specialized Hospital, Hawassa, Ethiopia; 4 Oral and Maxillofacial Pathology, Saveetha Dental College and Hospitals, Saveetha, Institute of Medical and Technical Sciences, Saveetha University, Chennai, Tamil Nadu 600077 India; 5 School of Dentistry, Faculty of Medical Sciences, The University of West Indies, St Augustine, Trinidad & Tobago; 6 Private Practitioner, Smile-Align Dental Clinic, Bangalore, India

**Keywords:** Primordial odontogenic tumor, Odontogenic tumor, Sub-Saharan Africa, Hybrid odontogenic lesion, WHO classification of odontogenic tumors, Definition proposal, New diagnostic criteria, Oral pathology

## Abstract

**Background:**

POT is a relatively newly described benign odontogenic tumor with very few cases registered to date. We present the 1st case of Primordial odontogenic tumor (POT) from Sub-Saharan Africa with unique clinicopathological features; also, this is the first case to report POT’s existence as a Hybrid Odontogenic lesion (HOL), with a pertinent review of the literature.

**Case presentation:**

This was a 17-year-old patient who presented with slow-growing, painless posterior mandibular swelling. The imaging revealed a well-defined, unilocular, expansile, lytic lesion with internal calcific foci surrounding an impacted #36, indicating a calcifying odontogenic cyst. The incisional biopsy revealed the presence of POT. The tumor was excised along with the involved tooth.

**Conclusion:**

POT is predominantly a non-aggressive and mostly affects the pediatric population. Hence, clinicians must be updated on all the aspects of this tumor to diagnose it appropriately and avoid any undue over—or under-treatment.

## Background

Introduced in 2014, ‘Primordial Odontogenic tumor’ (POT) was first incorporated into the WHO classification of Odontogenic tumors in 2017 under Benign odontogenic tumors of mixed epithelial and mesenchymal origin. At that time, only 7 cases of POT were known; astonishingly, even a decade later after the first time it was described, not more than 25 cases have been added [1, 2]. POT is a well-defined radiolucent lesion associated with an unerupted tooth with a marked preference for the posterior mandible. Histopathologically, it presents as a solid multilobulated mass composed of abundant loose fibromyxoid stroma with ectomesenchymal cells surfaced entirely by odontogenic epithelium resembling inner enamel epithelium with no evidence of dentine / hard tissue formation. Except for one case of recurrence, the majority of the cases have shown excellent prognosis [2–4].

Although, it is argued that POT is not capable of producing any hard tissue formation; of late, a few authors have reported some unfamiliar features of POT suggesting that the clinicopathological spectrum of this tumor may be much wider than what’s already known [5]. This may also alter our thoughts on the current understanding of histopathology of POT. From time to time, WHO revises its blue book ‘Tumor Nomenclature and Classification’ in the view to update the status of these tumors; this, primarily relies upon the new information acquired from fresh cases and studies conducted during the time interval between two editions. Therefore, the authors intend to build on the currently existing knowledge of POT by putting across the details of an intriguing case of this tumor.

### Case presentation

**Fig. 1:**
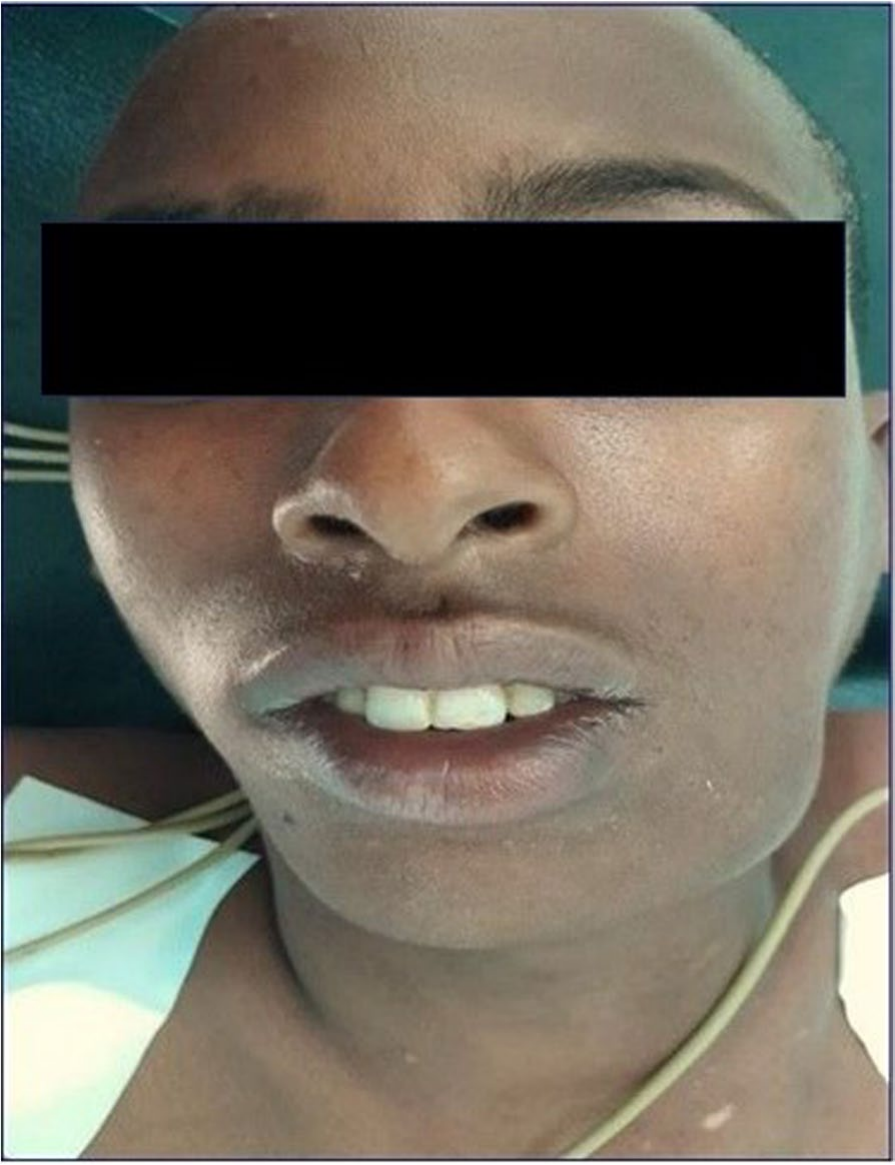
Clinical presentation of the patient. The extra-oral picture shows diffuse swelling over the left posterior mandibular region

**Fig. 2:**
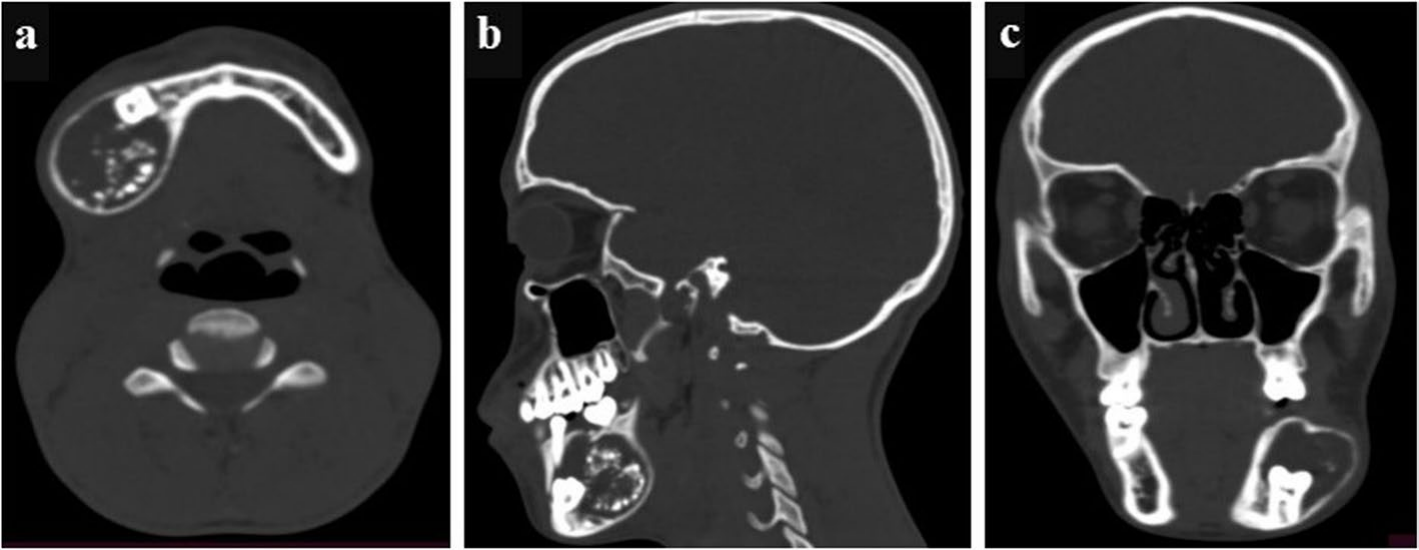
Radiographic features of the tumor. CT scan with contrast of the patient with (**a**) axial, **b** sagittal and (**c**) coronal views respectively demonstrate a well-defined unilocular radiolucency with multiple radio-opaque foci in the right mandible having a dentigerous type of association with the crown of a developing third molar; tooth displacement & root resorption w.r.t permanent second molar is seen. The buccal and lingual cortices are intact but show expansion

A 17-year-old male patient presented to the Oral & Maxillofacial Surgery Department of Hawassa University Comprehensive Specialized Hospital, Ethiopia with the chief complaint of a painless, slow-growing swelling in the left posterior mandible with a 1-year duration. The previous medical, dental, & family history were unremarkable. On extra-oral examination, there was a solitary swelling over the left angle of the mandible with diffuse borders and normal appearing overlying skin; no lymphadenopathy was identified (Fig. 1). Intra-orally, the swelling extended from 34 to 37 causing vestibular obliteration & cortical expansion; and 36 was missing in the oral cavity. It was mildly tender, bony hard on palpation with an intact overlying mucosa. The associated teeth were vital. FNAC was performed; it was suggestive of benign cystic contents. The Head & Neck CT scan revealed a well-defined, unilocular, expansile, lytic lesion with internal calcific foci measuring 4.2 × 3.3 × 3.3 cm (Fig. 2A) involving the left mandibular body surrounding an impacted 36 (Fig. 2B) and root divergence was noted w.r.t. 34 and 37 (Fig. 2C). Based on the clinical and radiographic features, a provisional diagnosis of calcifying odontogenic cyst was formed with the differential diagnosis of adenomatoid odontogenic tumor and calcifying epithelial odontogenic tumor. An incisional biopsy was performed and the histopathological diagnosis of a Primordial odontogenic tumor was given. Since POT is mostly known to be an innocuous neoplasm, enucleation of the lesion was performed with the surgical removal of 36; the specimen was sent for histopathological evaluation.

**Fig. 3:**
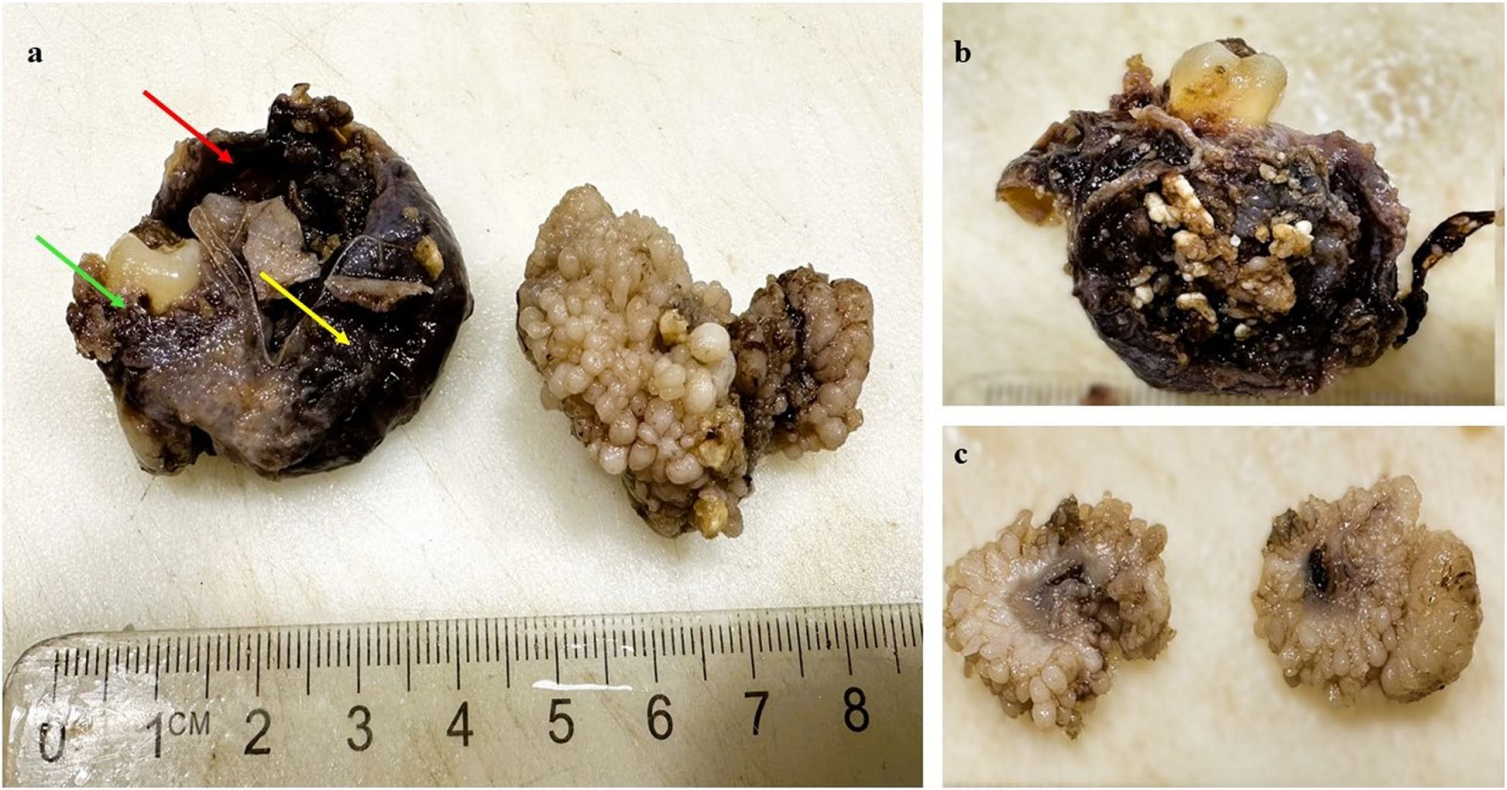
Gross picture of neoplastic tissue bits. (**a**) shows the first tissue bit with cystic (red arrow) and solid component (yellow arrow) attached to the neck of the tooth(green arrow); 3B: cut surface of 1st tissue bit with calcific nodules and 3C shows multilobated glistening gelatinous surface

Macroscopically, the specimen was received in two bits both measuring 4 × 3x3cm approximately. The first tissue bit was dark brown colored with both solid and cystic components encompassing an impacted 36, partly attached to the neck of the tooth (Fig. 3a). The surface was rough and irregular with firm consistency. Its cut section revealed multiple white round calcific nodules (Fig. 3b). The second tissue bit was yellowish-gray-white with a bossellated surface; the cut section was glistening white and firm to gelatinous consistency (Fig. 3c).

**Fig. 4:**
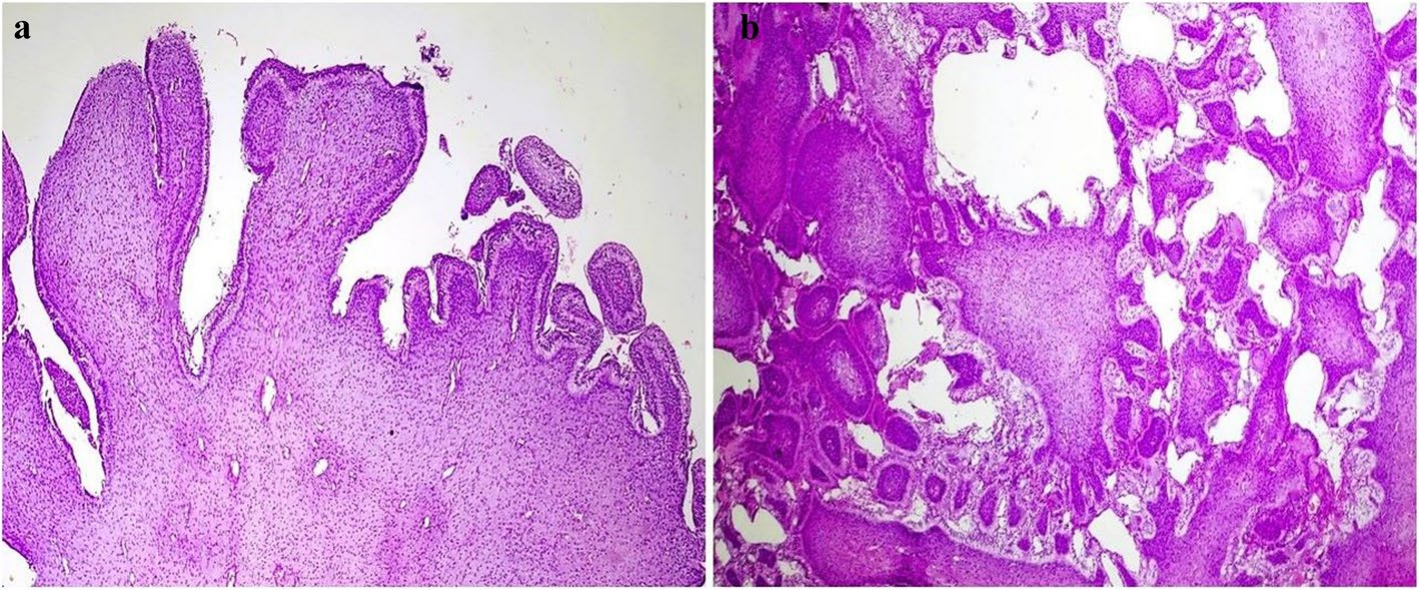
Microscopic features of the neoplasm (**a**) papillary arrangement of the neoplasm comprising amleoblastomatous lining overlying the fibro-myxoid bulk of the tumor; H&E scanner view (**b**) epithelial proliferations enveloping the fibro-myxoid tissue anastomosing with each other; H&E, 20 × view

**Fig. 5:**
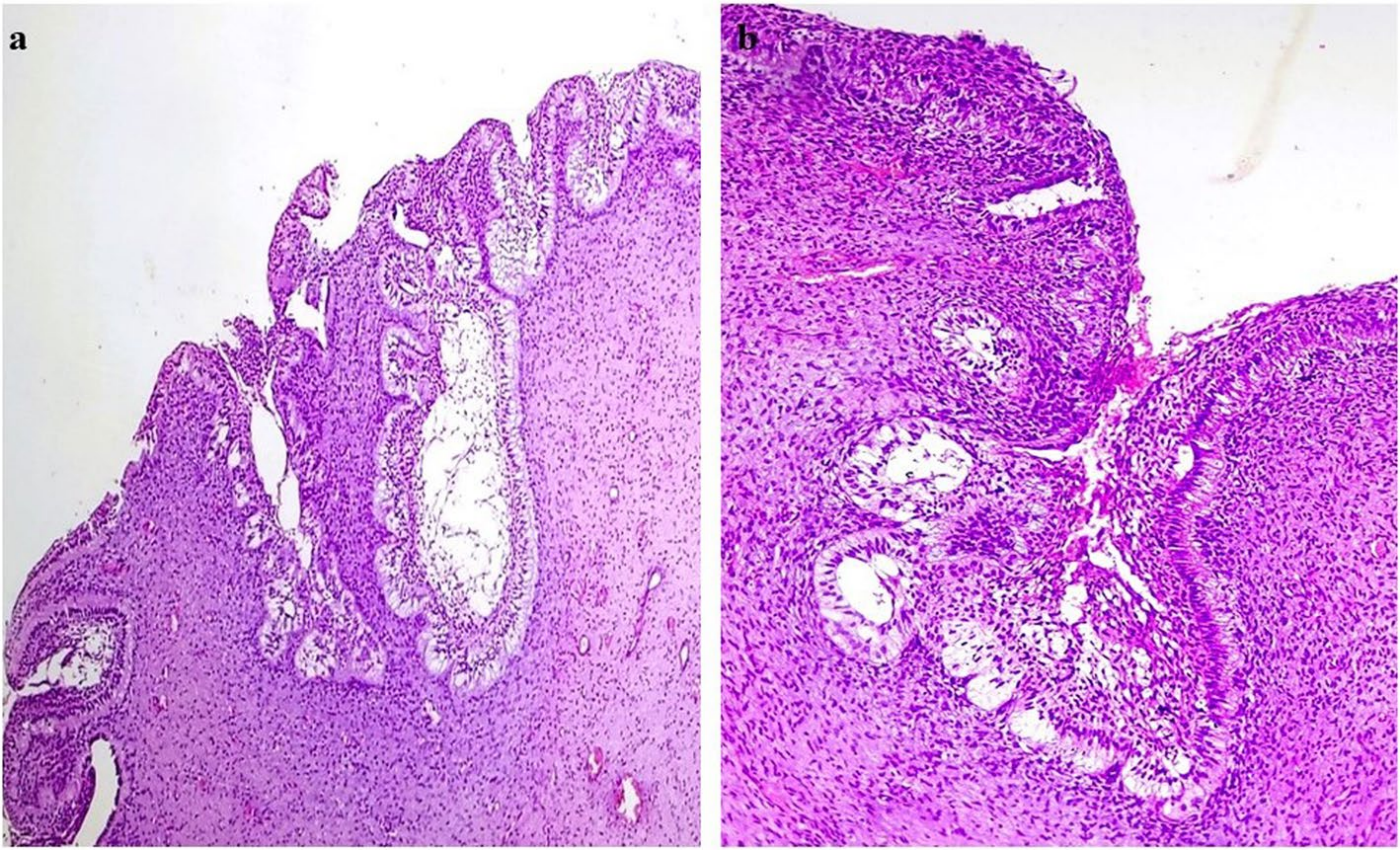
**a** & **b** Photomicrograph shows neoplastic epithelial lining invaginating into the underlying mesenchymal stroma in the form of cords and nests lined peripherally by tall columnar cells with hyperchromatic reversely polarized nuclei and central areas composed of loose arranged stellate reticulum simulating ameloblastoma cells; H&E, 10x & 20 × view respectively

**Fig. 6:**
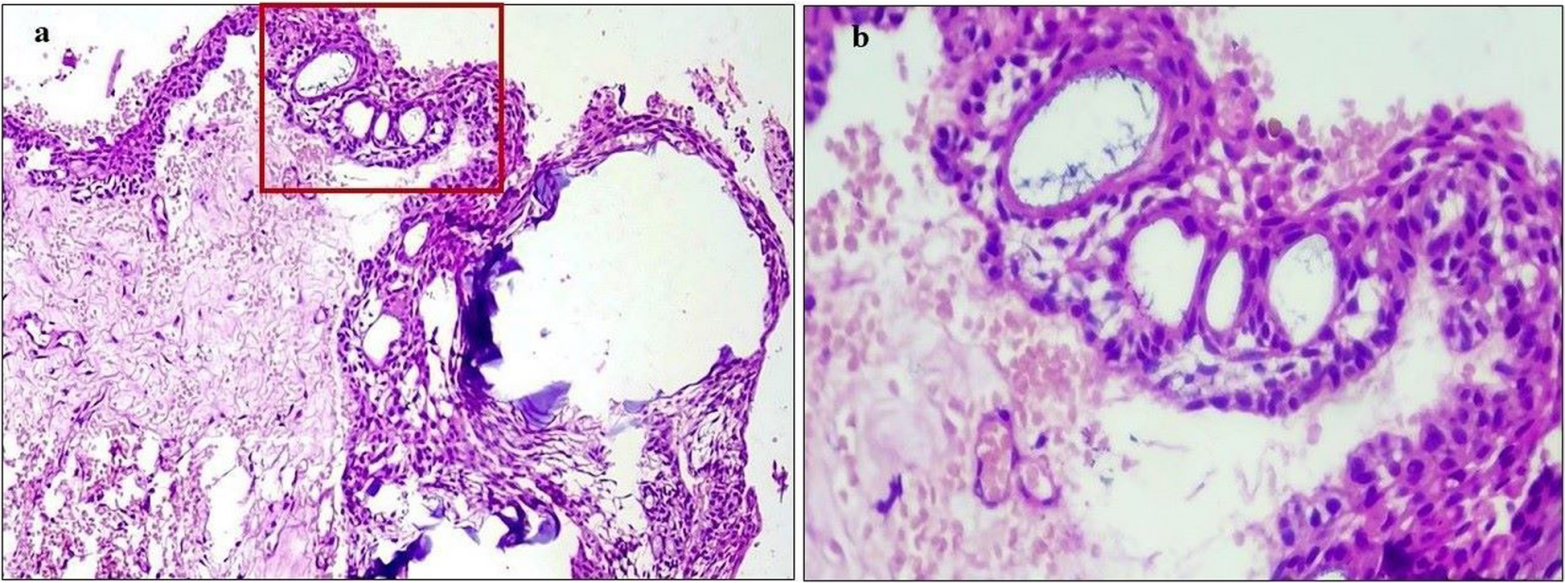
**a** & **b** Photomicrograph shows neoplastic epithelial lining containing duct-like structures with scarce basophilic secretory material surrounded by oval and round odontogenic cells, scattered clear cells; H&E, 10x & 40 × view respectively

Microscopically, the neoplasm was arranged in both broad papillary and flat pattern comprising surface epithelium made of tall columnar cells with hyperchromatic nuclei arranged in a palisading pattern with prominent superficial loosely arranged oval to spindle-shaped stellate reticulum-like cells anastomosing with adjacent epithelium (Fig. 4a & b) invaginating into the underlying fibro myxoid stroma of varying cellularity (Fig. 5a &b). A few clear cells and gland-like structures were also present (Fig. 6a &b).

**Fig. 7:**
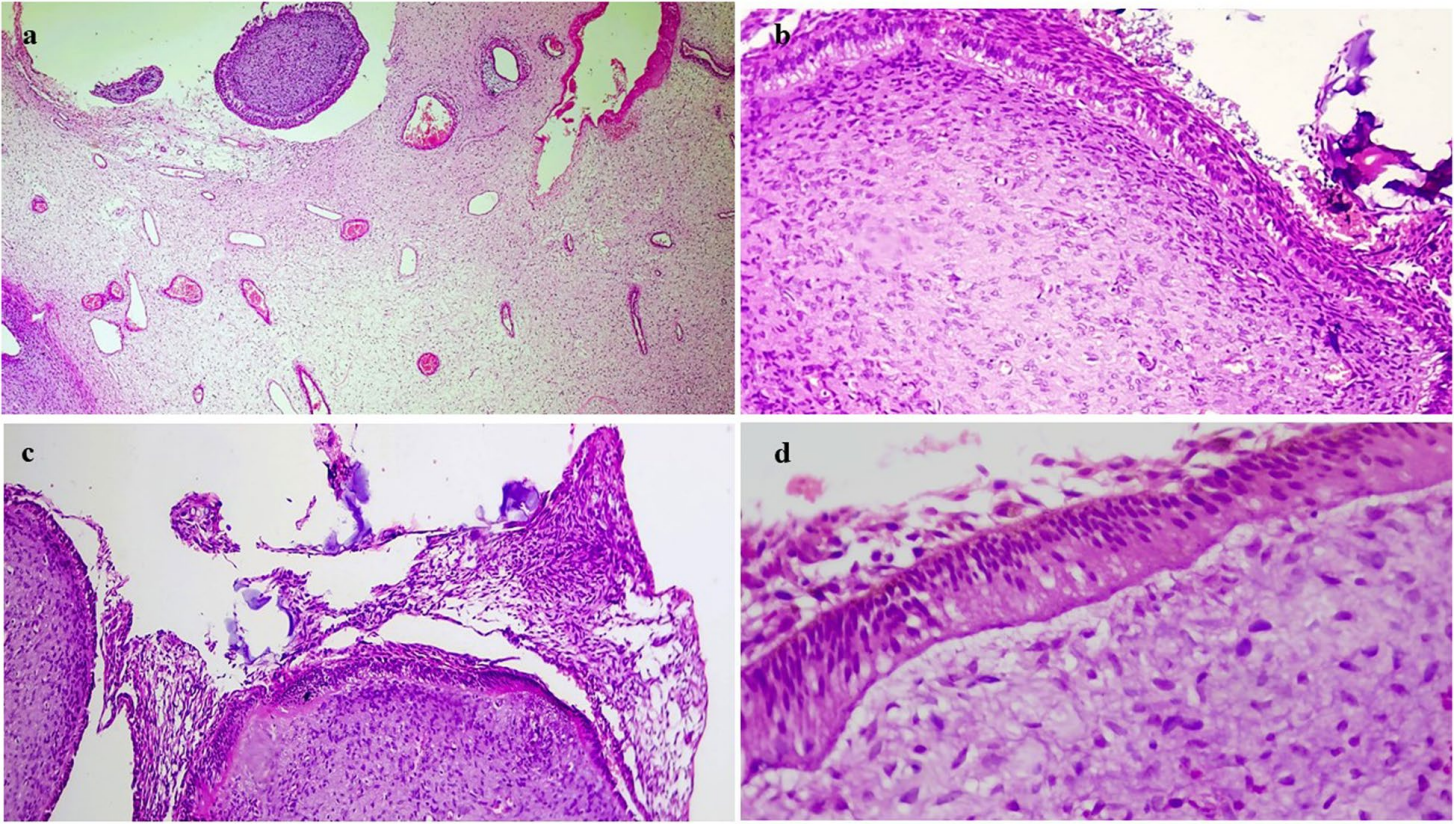
**a** Photomicrograph shows delicate fibromyxoid stroma with prominent vascularity; H&E, 5x (**b**) surface epithelium composed of tall columnar cells with hyperchromatic reversely polarized nuclei in a palisading pattern and superficial layers composed of flat stellate intermedium like cells and loosely placed stellate. shaped cells resembling stellate reticulum. A prominent sub-epithelial hypercellular cambium layer composed of oval -stellate dark staining mesenchymal cells is seen; H&E 20 × view. **c** a layer of eosinophilic material deposited at the epithelial-mesenchymal interface; H&E 20 × view (**d**) Focally, a cell- free zone is seen subjacent to the overlying epithelial cells; H&E 40 × view

**Fig. 8:**
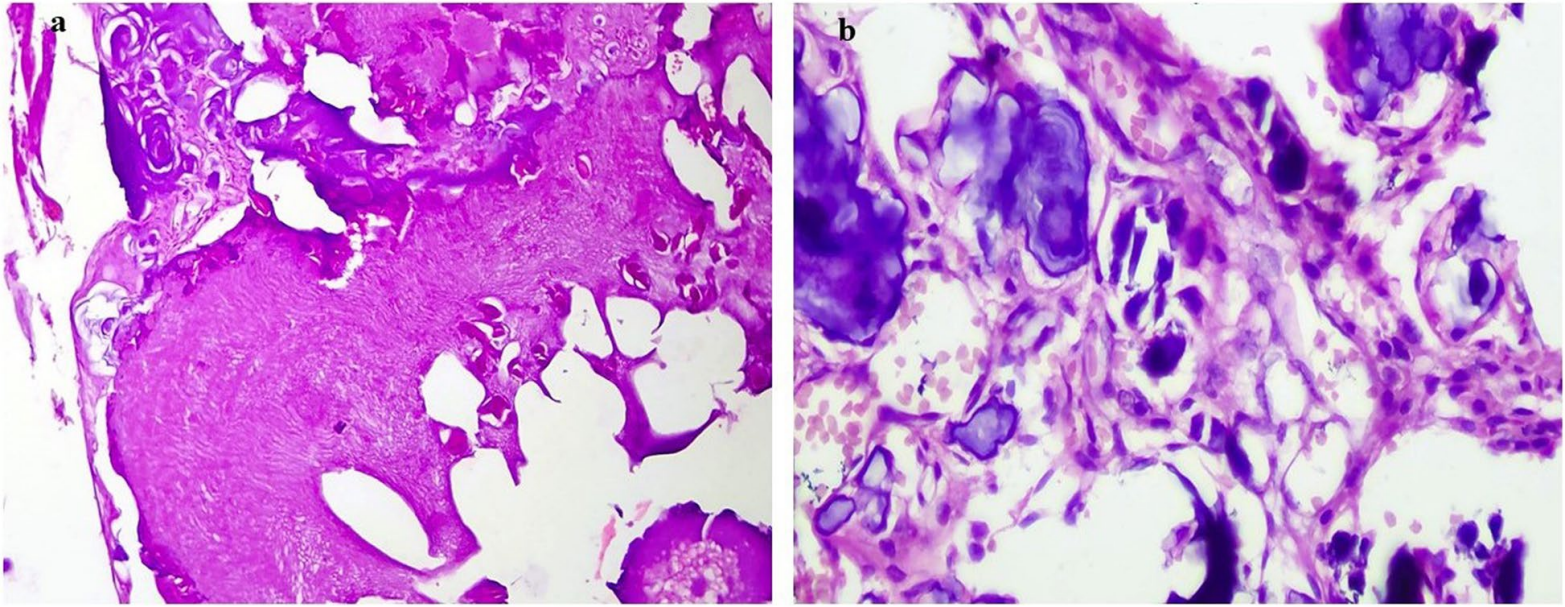
**a** & **b** The photomicrograph shows abundant calcification in the form of dentinoid and enameloid with prominent keyhole pattern of immature enamel along with the globular calcifications with concentric lamellations resembling Liesegang rings of CEOT; H&E, 20x & 40 × respectively

**Fig. 9:**
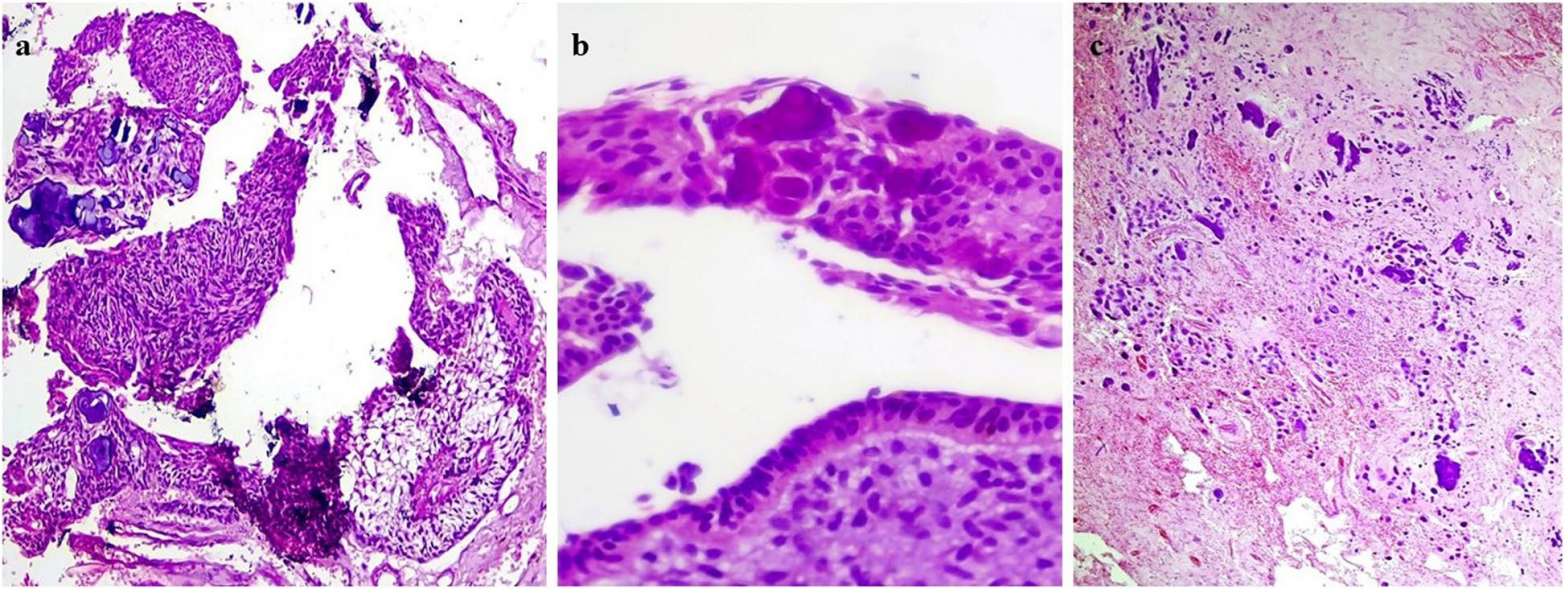
**a** & **b** Photomicrograph shows scattered intra- epithelial basophilic – amphophilic irregular and lamellar calcifications; H&E, 10x & 40 × view (**c**) dystrophic calcifications within the stroma; H&E, 5 × view

**Fig. 10:**
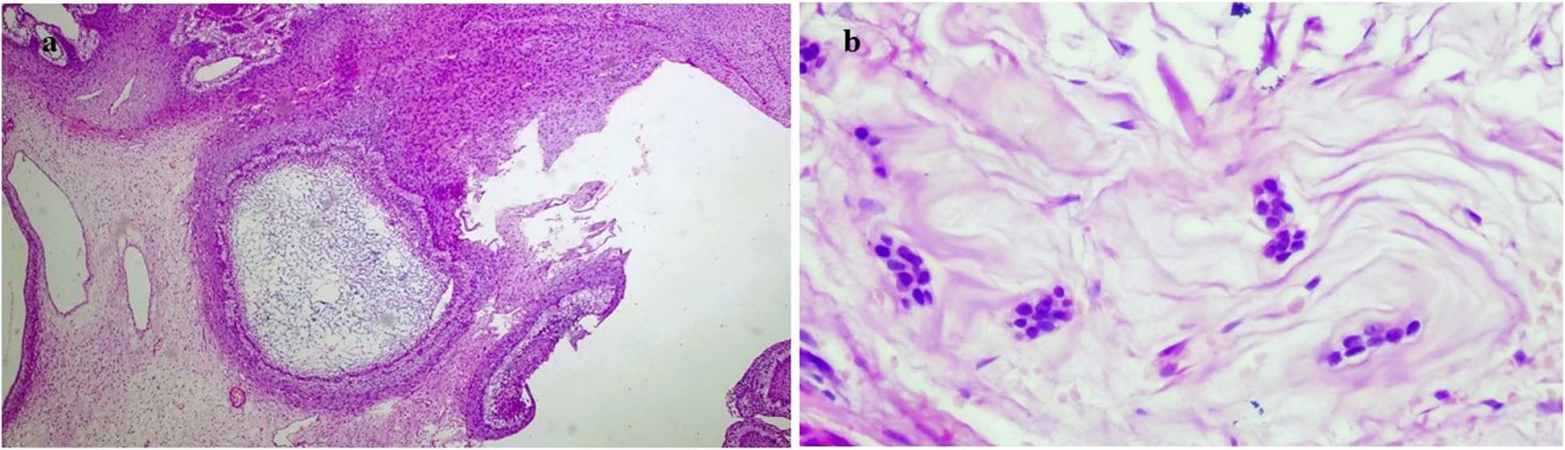
Additional features seen in the mesenchymal component of the neoplasm (**a**) The photomicrograph shows an abortive tooth germ like structure within the stroma; H&E 10 × view (**b**) odontogenic cell rests; H&E, 20 × view

The stroma was variably fibro-myxoid with prominent vasculature and, the sub-epithelial region consistently showed condensation of oval-spindle ecto-mesenchymal cells with a relatively hypocellular center (Fig. 7a & b). A band of eosinophilic secretory material was present focally below the tall columnar epithelial cells (Fig. 7c). Also, a cell-free zone was observed at the epithelial-stromal junction simulating the cell-free zone (Zone of Weil) present in the coronal pulp beneath the odontoblastic layer (Fig. 7d). There was an abundant deposition of enameloid exhibiting classical fish-scale pattern admixed with dentinoid material undergoing calcifications (Fig. 8a &b). Dystrophic calcifications were seen within the epithelium as well as the stroma (Fig. 9). An abortive tooth germ and odontogenic cell nests were also present in the stroma (Fig. 10).

**Fig. 11:**
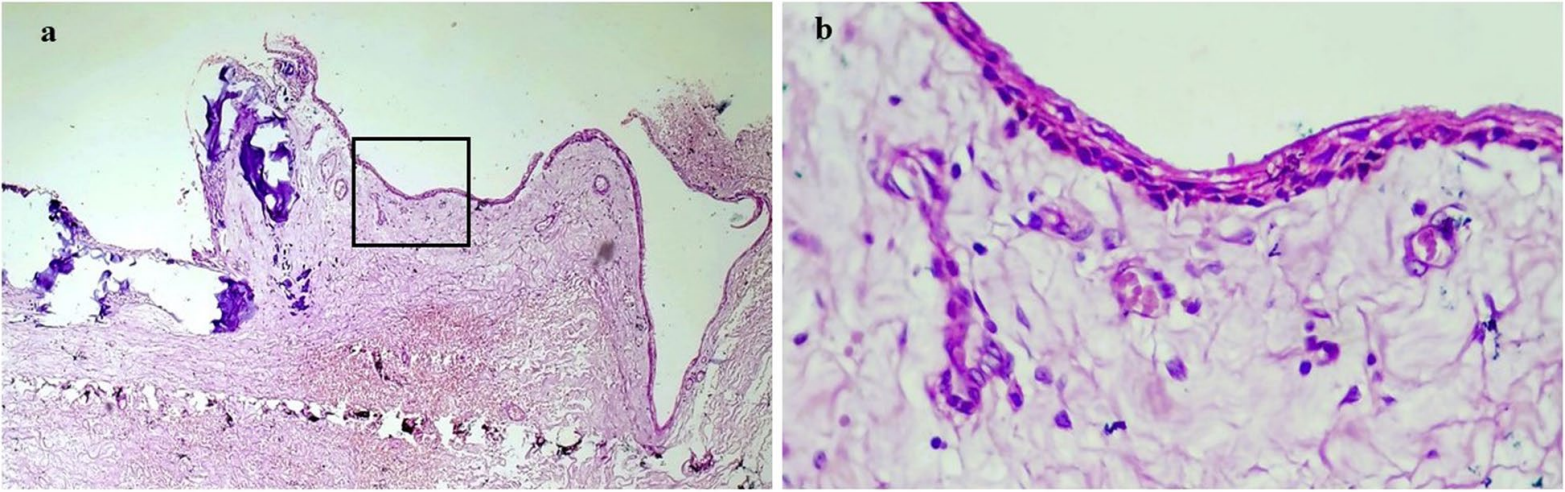
**a** the photomicrograph shows cystic lining on the right side becoming continuous with the neoplasm on the left side (Hybrid lesion); H&E, 10x (**b**) dentigerous cyst comprising of thin epithelial lining of 2–4 cell layers resembling reduced enamel epithelium; H&E, 40x

The features of the cystic component were consistent with that of a dentigerous cyst. It was composed of a thin 2–3 layered stratified squamous epithelial lining supported by a non-inflamed loose fibrous capsule comprising small nests of odontogenic cell rests and, this lining was in continuation with the tumor (Fig. 11). Hence, the diagnosis of a Hybrid odontogenic lesion (HOL) i.e. primordial odontogenic tumor occurring with a dentigerous cyst was established.

## Discussion

To date, 32 cases of POT have been described, out of which the majority came from Western countries; here, we report the very first case from Ethiopia, which is one of the 48 countries that constitute Sub-Saharan Africa [5]. Additionally, to the best of our knowledge, this is the first time that the occurrence of POT as a hybrid lesion has been reported in the world.

One of the objectives of this paper is to discuss and consolidate the most common clinicopathological findings of POT and converse over the more recent and novel findings which can further aid in a better understanding of this entity. The clinico-radiographic details of the previously reported cases along with the current case have been compiled in Table 1.

**Table 1:** Clinicopathological details of the previously documented cases of POT including the present one

Case no	Age	Sex	Site	Symptom	Duration	Radiographic	Radiographic dimension	Tooth involvement	Root resorption	Treatment	Recurrence	Follow -up	Reference no.
1	18	M	Left posterior mandible; angle	Asymptomatic	6 months	Well-defined, unilocular, radiolucency	45 × 40 mm	Enclosing 3rd molar crown	Yes	Enucleation	No	20 years	[1]
2	16	M	Left posterior mandible; angle	Asymptomatic	4 months	Well-defined, unilocular, radiolucency	55 × 50 mm	Enclosing 3rd molar crown	N/A	Enucleation	N/A	Lost to follow- up	[1]
3	16	M	Left posterior mandible; angle	Asymptomatic	1 year	Well-defined, unilocular, radiolucency	65 × 50 mm	Enclosing 3rd molar crown	No	Enucleation	No	10 years	[1]
4	3	F	Left posterior mandible; angle; ascending ramus	Asymptomatic	1 year 10 months	Well-defined, bilocular, radiolucency	90 × 70 mm		Yes	Enucleation	No	9 years	[1]
5	13	F	Left posterior mandible; angle; ascending ramus	Asymptomatic	4 months	Well-defined, bilocular, radiolucency	80 × 50 mm	Enclosing 3rd molar crown	Yes	Enucleation	No	3 years	[1]
6	3	F	Left posterior maxilla; sinus floor; tuberosity	Asymptomatic	3 months	Well-defined, unilocular, radiolucency	35 × 30 mm		N/A	Enucleation	No	6 months	[1]
7	19	M	Right posterior mandible	Asymptomatic	N/A	Well-defined, unilocular, radiolucency	N/A	Enclosing 3rd molar crown	Yes	Enucleation	No	7 months	[6]
8	15	F	Right posterior mandible; angle	Asymptomatic	N/A	Well-defined, multilocular, radiolucency	35 × 20 mm	Enclosing 3rd molar crown	Yes	Enucleation	No	3 months	[7]
9	18	M	Right posterior mandible	Asymptomatic	N/A	Well-defined, unilocular, radiolucency	17 × 12 mm	Enclosing 3rd molar crown	No	Curettage	No	20 months	[7]
10	8	F	Left posterior maxilla; sinus floor	Asymptomatic	N/A	Well-defined, unilocular, radiolucency	N/A		No	Enucleation	No	16 months	[8]
11	5	M	Right posterior mandible	Asymptomatic	N/A	Well-defined, unilocular, radiolucency	N/A		Yes	Enucleation	No	7 months	[9]
12	17	M	Left posterior mandible; angle	Asymptomatic	6 months	Well-defined, multilocular, radiolucency with scalloping	N/A	Enclosing 3rd molar crown	Yes	Enucleation	No	6 months	[10]
13	2	M	Right posterior mandible; angle; ascending ramus	Painful	2 months	Well-defined, multilocular, radiolucency	30 × 40 mm		N/A	Enucleation	No	2 years	[11]
14	4	M	Left posterior mandible	Asymptomatic	8 months	Well-defined, unilocular, radiolucency	30 × 20 mm		Yes	Enucleation	N/A	Lost to follow-up	[12]
15	13	F	Left posterior mandible; angle; ascending ramus	Asymptomatic	3 months	Well-defined, unilocular, radiolucency	N/A	Enclosing 3rd molar crown	Yes	Enucleation	N/A	N/A	[13]
16	Pa	M	Left posterior mandible; angle; ascending ramus	Asymptomatic	N/A	Well-defined, unilocular, mixed radiolucent-radiopaque	22 × 20 mm	Missing 17	No	Enucleation	N/A	N/A	[14]
17	10	M	Left posterior mandible	Asymptomatic	N/A	Well-defined, unilocular, radiolucency	5 × 5x5mm	Mesiolingual to the root of 34	No	Enucleation	No	12 months	[15]
18	17	F	Left posterior mandible; angle; ascending ramus	Asymptomatic	1 month	Well-defined, unilocular, mixed radiolucent-radiopaque	34 × 25 mm	Enclosing 3rd molar crown	No	Enucleation	No	18 months	[16]
19	16	M	Right posterior mandible	Asymptomatic	N/A	Multilocular- Radiolucent	20 × 15 mm	Supernumerary	No	Enucleation	No	1 year	[17]
20	6	N/A	Left front maxilla	Asymptomatic	N/A	Unilocular radiolucency with odontoma like structure	N/A	Impacted 21	N/A	enucleation	No	10 years	[18]
21	13	M	Left front maxilla	Asymptomatic	6 months	Well defined unilocular radiolucency	32 × 31 mm	Impacted 23	N/A	Excision N/A	N/A	N/A	[19]
22	8	M	Left maxilla	Asymptomatic	3 months	Well-defined unilocular mixed radiopaque-radiolucency	30 × 30 mm	Impacted 24	Yes	Curettage	N/A	N/A	[20]
23	2	M	Right maxilla	Asymptomatic	2 weeks	Well-defined unilocular radiolucency	25 × 22x21mm	Unerupted 54	N/A	enucleation	No	11 months	[5]
24	12	F	Right maxilla	Asymptomatic	2 months	Well-defined unilocular radiolucency	35 × 25x25mm	N/A	Yes	Excision	No	13 years	[5]
25	3	M	Right maxillary gingiva	Asymptomatic	2 weeks	Mixed radiopaque- radiolucen2cy	7 × 7 mm	Impacted 2nd primary molar	N/A	excision	No	3 years	[21]
26	3	F	Left posterior mandible	Painful	N/A	Multilocular radiolucency extending upwards into the posterior zygomatic arch, infra‑temporal fossa as well as the orbital floor	N/A	Developing teeth	N/A	Incisional	N/A	N/A	[22]
27	10	M	Right maxilla	Asymptomatic	N/A	Well defined unilocular mixed radiopaque-radiolucency	N/A	Distal root of unerupted 2nd deciduous molar	No	enucleation	No	30 months	[23]
28	14	M	Right maxilla	Asymptomatic	4 months	Well-defined, unilocular radiolucency	20 × 15 mm	Impacted Central incisor and canine	N/A	excision	No	3 years	[24]
29	26	M	Left mandible	Asymptomatic	N/A	Well-defined unilocular mixed radiopaque radiolucency	N/A	Impacted third molar	N/A	Surgical removal	No	5 month	[4]
30	14	M	Left posterior mandible	Asymptomatic	N/A	Well-defined unilocular radiolucency	N/A	Impacted 2nd molar	Yes	N/A	No	11 months	[25]
31	12	F	Right mandible; mid-body	Asymptomatic	8 months	Well-defined unilocular radiolucency	N/A	Impacted 2nd pre-molar	Yes	Surgical removal	No	15 months	[26]
32	13	F	Left mandible	Asymptomatic	N/A	Well-defined unilocular radiolucency	N/A	Unerupted 3rd molar	N/A	enucleation	No	60 months	[26]
33	17	M	Left mandible body	Asymptomatic	1 year	Well-defined unilocular mixed radiopaque-radiolucent lesion	42 × 33x33mm	Impacted 1st molar	No	Enucleation with peripheral osteotomy	No		Present case

Table 1 Clinicopathological details of the previously documented cases of POT including the present one. (Attached as a separate file i.e. under ‘Tables’ section). It predominantly occurs at a young age with the patient age spanning from 2- 26 years old with a mean age of 11.43 years. The tumor has a male predilection (M: F: 1.9:1) and a site preponderance for posterior mandible (69.69%) followed by posterior maxilla; interestingly, anterior segment involvement has been seen only in maxillary cases. Albeit, the current literature comprises almost 30 confirmed cases of POT, the real incidence of POT still cannot be ascertained because of the high possibility of this neoplasm being misdiagnosed as some other entity in the past. Furthermore, a few systematic reviews chose to exclude the cases of POT with new features as these have not been recognized by WHO yet; but, could get accepted in the future [12, 26].

The majority of cases including this one, presented as a slow-growing, asymptomatic intra-bony swelling (except one gingival case) with/without cortical expansion observed during the later course of the disease [21]. Although most cases followed an indolent course, Zeng et al. and Almazyad A et al. reported cases with relatively aggressive behavior causing mandibular perforation followed by recurrence in the latter [3, 5]. It most often presented as a well-defined radiolucent lesion with a unilocular appearance in 81.8%, multilocular in 12%, and bilocular in 6% of the cases, mostly associated with an impacted / unerupted tooth from either deciduous, permanent, or supernumerary dentition. In this case, the tumor appeared to envelop the mandibular 1st molar (Sun et al., type B category) [15]. Size-wise, it exhibited great variability ranging from 90 × 70 to 5 × 5 mm with a mean radiographic size of 35.9 × 29 mm. Variable degree of tooth displacement and root resorption was also noted in a few cases. Significantly, a calcified foci was also observed in this case which is an uncommon feature of POT and was observed in only 6 cases. Hence, a differential diagnosis of POT should not only be restricted to radiolucent lesions but also a mixed radiolucent-opaque lesions with/ without cystic component.

Grossly, most POTs appeared as a solid, multilobated mass with a shiny surface and gelatinous consistency. Additionally, cystic changes were seen in the present case which is an unusual finding and has only been found in three other cases reported by Tomasz et al. [17]. Although well-delineated from the surrounding structures, the presence of fibrous capsules is not common in POT (24%). Microscopically characterized as a biphasic neoplasm, it comprises a distinctive superficial layer of cuboidal- columnar odontogenic cells along with an underlying loose myxoid to minimally fibrous ecto-mesenchymal stroma (akin to dental papilla) containing spindle stellate shaped cells which constitutes the bulk of the tumor; both these features are seen invariably in 100% cases.

However, a few variations in the superficial epithelial layer have been reported in the last few years. The surface epithelium, in this case, showed a papillary configuration (6 /33) (due to the differential growth rate of the neoplastic cells) as well as a double-layered invagination (11/33) into the underlying stroma with microcystic changes and a significant quantity of dystrophic calcifications (11/33). Similar to the case of Zeng et al. and Asma et al., a rich proliferative layer of stellate-reticulum-like cells was also seen superficial to the columnar cells anastomosing with the adjacent epithelium to form a plexiform pattern; this is in contrast to its sparse distribution when seen in other cases. (17/33). A prominent juxta-epithelial cell-rich cambium layer was also present as it was in almost half of the other cases accompanied by rudimentary enamel organ-like structures which is otherwise a rare finding. One of the most noteworthy features of this case was the copious quantities of calcification scattered throughout the neoplastic stroma. Another striking element of this case was the first-time presentation of such an extensive enamel matrix exhibiting the classical keyhole pattern along with surplus dentinoid. Enameloid and dentinoid aggregation have been among the newest features seen in five and two other cases respectively.

Last but not least, the hybrid nature of this tumor is another riveting element of this case. Although rarely, dentigerous cysts have been known to both a) transform into neoplasms and, b) coevolve with other lesions [27, 28]. Notably, POT and DC share some common features which include their odontogenic nature, age predilection for 1st and 2nd decade, site preference for the posterior mandible, association with an unerupted tooth, and benign and indolent behavior with a low rate of recurrence [25]. This is the world’s first-ever case to document POT and DC co-existing in the same space. Now having said that, we must not close our doors to the potentiality of DC giving rise to POT.

As evident, our case presented with rare radiographic and histological features while the clinical and demographic features were similar to the ones reported previously. The histological diagnosis should not be difficult given the peculiar features in this neoplasm; however, some might find it difficult to differentiate it from a few entities in Table 2.

**Table 2:** Histopathological differential diagnosis of POT with differentiating features

S.No	Pathological entity	Differentiating points	Reference
1.	Dental papilla (DP)	• Surface layer of odontoblasts absent in POT • Surface layer of inner enamel epithelium & stellate reticulum-like cells seen in POT • Calcifications /enameloid/dentinoid material may be seen in POT absent in dental papilla • Smaller size of DP • Negative for cytokeratins	[29]
2.	Odontogenic myxoma (OM)	• Surface layer of inner enamel epithelium seen in POT, absent in OM • Higher mesenchymal cellularity in POT • Radiographic Honey-comb appearance of OM • OM is rarely associated with an impacted tooth	[2]
3.	Ameloblastic fibroma (AF)	• Surface layer of inner enamel epithelium seen in POT, is absent in AF • mesenchymal cells are more cellular mostly abundant ameloblastic epithelial cords & nests in AF stroma	[2, 22]
4.	Ameloblastic fibro-odontoma (AFO)	• Surface layer of inner enamel epithelium seen in POT, absent in AFO • mostly abundant ameloblastic epithelial cords & nests in AFO stroma	[2, 22]
5.	Developing Odontoma (OD)	• Intraepithelial eosinophilic material/calcification is not a feature of developing odontoma • Dentine and enamel matrix is formed at the interface between epithelium and mesenchyme in DO	[6]
6.	Archegonous cystic odontoma	• Archegonous cystic odontoma is a cystic lesion with dentinoid deposition, • is present in place of a tooth and not in a dentigerous association • does not resemble this solid tumor • no evidence of ameloblastomatous ‘‘reverse polarization’’ of nuclei	[30]

Thus far, most cases have been treated with enucleation (63.6%) followed by simple surgical excision (18.1%) and curettage (6%); however, two cases were treated more aggressively. Partial mandibulectomy was performed for one due to its initial histological diagnosis of odontogenic myxoma while hemi mandibulectomy was done for the sole case of recurrent POT which was initially enucleated 4 years ago. None of the other cases have shown recurrence with the follow-up period ranging from six months – 20 years. The updated diagnosis of a hybrid lesion didn’t warrant any further treatment. The patient has been on follow-up with no signs of recurrence for two years.

Although immunohistochemical and genetic tests are non-essential for diagnosing POT, they are certainly crucial for understanding the true nature of the neoplastic components. In an attempt to decipher the true pathogenesis of this neoplasm, a variety of diagnostic analyses have been performed on POT tissues over time. In normal odontogenesis, CK-19 is invariably expressed in the pre-ameloblasts and is slowly replaced by CK-14 as it matures into secretory ameloblasts (cap-to-bell stage transition). Similar cytokeratin expression has been seen unvaryingly in the epithelial layers of POT suggesting that the epithelial cells could inherit either pre-ameloblastic or ameloblastic properties [31]. The ameloblastic nature of the neoplastic cells was also confirmed by Mikami et al. as they revealed the focal presence of amelogenin and ameloblastin as well as enamel protein coding genes i.e. Amelx, Ambn and Enam in the surface epithelium. Moreover, amelogenin was also detected in the calcified particles and non‐keratinized squamous cells surrounding them, suggesting that the calcifications resembled enamel and amelogenin might contribute to its production in POT. Apart from the enamel coding genes, mRNA of major dentin coding genes i.e. Colla1, Dspp and Dmp-1 were highly expressed in both epithelial and mesenchymal components which are normally seen in both pre-ameloblasts & pre-odontoblasts [32, 33]. This justifies the presence of both enamel and dentin in the present case.

Both Vimentin and Syndecan-1 exhibited inconsistent and focal presence in the neoplastic epithelium contrary to a constant strong expression observed in mesenchymal components, especially in the sub-epithelial condensation. This not only supports the primordial origin of the tumor but also reflects its ability to demonstrate epithelial-mesenchymal transitions/interactions essential for tissue differentiation and morphogenesis pre-odontoblastic differentiation, epithelial differentiation as well as mesenchymal cell condensation & proliferation [31, 34].

Also, POT has been shown to unevenly express some additional markers like Glut-1, MOC-31, and Caveolin-1. Galectin-3, PITX2, p53, Bax, Bcl-2, Survivin, and PTEN in focal areas, furthermore suggesting the diverse nature of POT in terms of its cell maturity and stages of differentiation [26]. The indolent clinical behavior of the tumor along with a low index of Ki-67 (< 4%) of neoplastic epitheliums suggests its innocuous nature [1, 7, 15, 34]. However, there have been two cases that exhibited a destructive disease course followed by a recurrence which could be associated with a higher Ki-67 (20–30%) in the sub-epithelial neoplastic stroma. This warrants the study of more cases until the true biological course of POT can be known [1, 3, 5, 31].

POT, beyond any doubt, is a novel tumor with distinct microscopic features that differ in its genetic pathogenesis from other odontogenic tumors The differentiation stage of POT bears striking semblance to an early tooth germ anywhere between the 10th and 20th week of its development; thereby mitigating its potential to produce hard tissues (enamel, dentin) [1, 32]. The criteria laid down by the latest 5th edition of WHO (2022) for its diagnosis are still based upon the findings of seven cases described by Mosqueda-Taylor et al. ten years back [3, 35, 36]. Although the data at the time was adequate for the initial recognition of this peculiar tumor as a new entity, it cannot possibly represent the entire spectrum of this tumor. The findings of the further added cases remain excluded which can lead to a misdiagnosis or a missed diagnosis! For example, the presence of enamel, dentin, and other calcifications in the tumor that has been validated by relevant immunohistochemical and genetic studies; remains to be acknowledged yet! However, we can all agree that it would still require a lot more cases to understand its full spectrum. Here, we have compiled the data of thirty-two additional cases including the present one, and focused on the new features identified in our case and other cases. In light of the recent studies, we recommend updated diagnostic criteria for the tumor and suggest that the following novel features be added to the existing WHO diagnostic criteria:

A. Its occurrence as a mixed radiolucent–radiopaque lesion.
B. Stellate reticulum-like areas superficial to the tall columnar cells.
C. Presence of dystrophic calcification within the epithelium and stroma including lisegang ring lamellar bodies.
D. Presence of odontogenic hard tissue including enameloid, dentin/ dentinoid, and cementum

Also, a new definition can be considered in the future i.e. Primordial odontogenic tumor is a tumor composed of variably cellular loose fibrous tissue with areas similar to the dental papilla, entirely surrounded by cuboidal to columnar epithelium resembling the internal epithelium of the enamel organ with or without hard tissue formation.

## Conclusion

Although most POTs are associated with an unerupted tooth, the possibility of its occurrence otherwise should also be kept in mind. It should be considered as a differential not just for a unilocular radiolucent lesion but also for a multilocular mixed lesion with or without a cystic component. This case report discusses an array of new-fangled features noticed in POT along with the supporting molecular & genomic evidence. This will guide the clinicians and pathologists to diagnose this tumor accurately, facilitating appropriate patient management.

## Data Availability

The datasets used and/or analyzed during the current study are available from the corresponding author on reasonable request.

## Abbreviations

POT: Primordial odontogenic tumor
WHO: World Health’s Organization
DC: Dentigerous cyst
HOL: Hybrid odontogenic lesion
Colla: Collagen type 1
Dspp: Dentin sialophosphoprotein
Dmp1: Dentin matrix protein1
IHC: Immunohistochemistry
CK: Cytokeratins
PCNA: Proliferating cell nuclear antigen
Glut-1: Glucose transporter 1
PITX2: Paired like homeodomain transcription factor 2

## Data availability

No datasets were generated or analysed during the current study.
